# 4-Methyl­anilinium 3,5-dinitro­benzoate

**DOI:** 10.1107/S1600536810018441

**Published:** 2010-06-05

**Authors:** Rui-jun Xu

**Affiliations:** a Ordered Matter Science Research Center, College of Chemistry and Chemical Engineering, Southeast University, Nanjing 210096, People’s Republic of China

## Abstract

The crystal structure of the title compound, C_7_H_10_N^+^·C_7_H_3_N_2_O_6_^−^, displays N—H⋯O hydrogen bonding between the ammonium groups and the O atoms of the 3,5-dinitro­benzoate anions. Inter­molecular C—H⋯O inter­actions further stabilize the packing. An O atom of each of the nitro groups is disordered over two sites with site occupancy factors of 0.59 (5) and 0.41 (6).

## Related literature

For dielectric–ferroelectric properties, see: Li *et al.* (2008 ▶). For a related structure, see: Basaran *et al.* (1991 ▶).
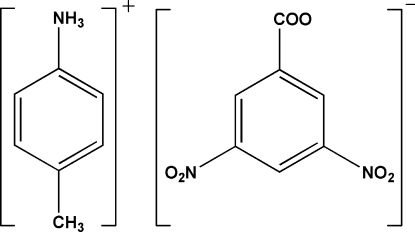


## Experimental

### 

#### Crystal data

C_7_H_10_N^+^·C_7_H_3_N_2_O_6_^−^
*M* *_r_* = 319.27
Orthorhombic, 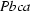

*a* = 19.790 (4) Å
*b* = 7.2380 (14) Å
*c* = 20.473 (4) Å
*V* = 2932.5 (10) Å^3^
*Z* = 8
Mo *K*α radiation
μ = 0.12 mm^−1^
*T* = 293 K
0.2 × 0.2 × 0.2 mm

#### Data collection

Rigaku Mercury2 diffractometer
Absorption correction: multi-scan (*CrystalClear*; Rigaku, 2005 ▶) *T*_min_ = 0.978, *T*_max_ = 0.978
28284 measured reflections
3360 independent reflections
2368 reflections with *I* > 2.0 σ(*I*)
*R*_int_ = 0.078

#### Refinement

*R*[*F* ^2^ > 2σ(*F* ^2^)] = 0.054
*wR*(*F* ^2^) = 0.159
*S* = 0.96
3360 reflections
230 parameters
H-atom parameters constrained
Δρ_max_ = 0.21 e Å^−3^
Δρ_min_ = −0.22 e Å^−3^

### 

Data collection: *CrystalClear* (Rigaku, 2005 ▶); cell refinement: *CrystalClear*; data reduction: *CrystalClear*; program(s) used to solve structure: *SHELXS97* (Sheldrick, 2008 ▶); program(s) used to refine structure: *SHELXL97* (Sheldrick, 2008 ▶); molecular graphics: *SHELXTL* (Sheldrick, 2008 ▶); software used to prepare material for publication: *PRPKAPPA* (Ferguson, 1999 ▶).

## Supplementary Material

Crystal structure: contains datablocks I, New_Global_Publ_Block. DOI: 10.1107/S1600536810018441/pv2280sup1.cif

Structure factors: contains datablocks I. DOI: 10.1107/S1600536810018441/pv2280Isup2.hkl

Additional supplementary materials:  crystallographic information; 3D view; checkCIF report

## Figures and Tables

**Table 1:** Hydrogen-bond geometry (Å, °)

*D*—H⋯*A*	*D*—H	H⋯*A*	*D*⋯*A*	*D*—H⋯*A*
N1—H1*A*⋯O2^i^	0.89	1.94	2.803 (2)	163
N1—H1*B*⋯O1	0.89	1.90	2.761 (2)	163
N1—H1*C*⋯O2^ii^	0.89	2.22	3.045 (2)	153
N1—H1*C*⋯O1^ii^	0.89	2.24	3.030 (2)	147
C3—H3⋯O1^ii^	0.93	2.59	3.344 (3)	138
C13—H13⋯O3^iii^	0.93	2.43	3.351 (7)	173

Symmetry codes: (i) 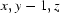; (ii) 
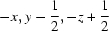
; (iii) 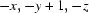.

**Fig. 1.**
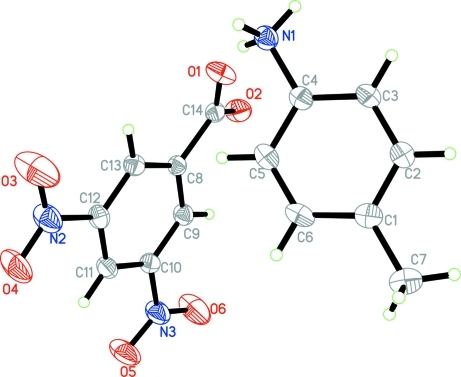
The molecular structure of the title compound, showing the atomic numbering scheme. Displacement ellipsoids are drawn at the 30% probability level. O3' and O6' representing the smaller fractions of the disordered atoms have been excluded.

**Fig. 2.**
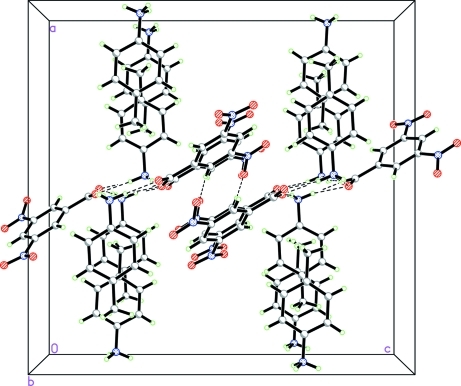
A view of the packing of the title compound, stacking along the b-axis. Dashed lines indicate hydrogen bonds.

## supplementary crystallographic information

### Comment

Probing the dielectric-ferroelectric properties of organic ligands (Li *et al.*, 2008), the title compound has been prepared in our laboratory. In this article, the preparation and crystal structure of the title compound have been presented. A related structure, that of 4-tethylanilinium dichloroacetate, has been reported previously (Basaran *et al.*, 1991).

The asymmetric unit of the title compound composes of a (CH_3_—C_6_H_4_—NH_3_^+^) cation and an (2(NO_2_)-C_6_H_3_—COO^-^) anion (Fig. 1). The strong N—H···O hydrogen bonds involving H1A and H1B (N1···O2 2.803 (2) and N1···O1 2.761 (2) Å) and the bifurcated hydrogen bonds involving H1C (N1···O2 3.045 (2) and N1···O1 3.030 (2) Å) are beneficial to the stability of the crystal structure (Fig. 2 and Tab. 1). Hydrogen bonds of the type C—H···O further stabilize the crystal structure.

### Experimental

The title compound was obtained by the addition of 3,5-dinitrobenzoic acid (4.66 g, 0.022 mol) to a solution of 4-methylaniline (2.14 g, 0.02 mol) in ethanol, in the stoichiometric ratio 1.1:1. After two weeks, good quality single crystals were obtained by slow evaporation.

### Refinement

O3 and O6 atoms of the nitro groups were disordered over two sites with site occupancy factors 0.59 (5) and 0.41 (6). Positional parameters of all the H atoms were calculated geometrically and the H atoms were set to ride on the C and N atoms to which they are bonded with N—H = 0.89 Å and C—H = 0.93 and 0.96 Å for aryl and methyl H-atoms, respectively, with *U*_iso_(H) = 1.2*U*_eq_(C or N).

### Crystal data

**Table:**

C_7_H_10_N^+^·C_7_H_3_N_2_O_6_^−^	*F*(000) = 1328
*M**_r_* = 319.27	*D*_x_ = 1.446 Mg m^−^^3^
Orthorhombic, *P**b**c**a*	Mo *K*α radiation, λ = 0.71073 Å
Hall symbol: -P 2ac 2ab	Cell parameters from 11511 reflections
*a* = 19.790 (4) Å	θ = 3.0–27.5°
*b* = 7.2380 (14) Å	µ = 0.12 mm^−^^1^
*c* = 20.473 (4) Å	*T* = 293 K
*V* = 2932.5 (10) Å^3^	Prism, colorless
*Z* = 8	0.2 × 0.2 × 0.2 mm

### Data collection

**Table:**

Rigaku Mercury2 diffractometer	3360 independent reflections
Radiation source: fine-focus sealed tube	2368 reflections with *I* > 2.0 σ(*I*)
graphite	*R*_int_ = 0.078
Detector resolution: 13.6612 pixels mm^-1^	θ_max_ = 27.5°, θ_min_ = 3.2°
CCD_Profile_fitting scans	*h* = −25→25
Absorption correction: multi-scan (*CrystalClear*; Rigaku, 2005)	*k* = −9→9
*T*_min_ = 0.978, *T*_max_ = 0.978	*l* = −26→26
28284 measured reflections	

### Refinement

**Table:**

Refinement on *F*^2^	Secondary atom site location: difference Fourier map
Least-squares matrix: full	Hydrogen site location: inferred from neighbouring sites
*R*[*F*^2^ > 2σ(*F*^2^)] = 0.054	H-atom parameters constrained
*wR*(*F*^2^) = 0.159	*w* = 1/[σ^2^(*F*_o_^2^) + (0.0824*P*)^2^ + 0.969*P*] where *P* = (*F*_o_^2^ + 2*F*_c_^2^)/3
*S* = 0.96	(Δ/σ)_max_ = 0.026
3360 reflections	Δρ_max_ = 0.21 e Å^−^^3^
230 parameters	Δρ_min_ = −0.22 e Å^−^^3^
0 restraints	Extinction correction: *SHELXL*, Fc^*^=kFc[1+0.001xFc^2^λ^3^/sin(2θ)]^-1/4^
Primary atom site location: structure-invariant direct methods	Extinction coefficient: 0.0065 (11)

### Special details

**Table:**

Geometry. All esds (except the esd in the dihedral angle between two l.s. planes) are estimated using the full covariance matrix. The cell esds are taken into account individually in the estimation of esds in distances, angles and torsion angles; correlations between esds in cell parameters are only used when they are defined by crystal symmetry. An approximate (isotropic) treatment of cell esds is used for estimating esds involving l.s. planes.
Refinement. Refinement of *F*^2^ against ALL reflections. The weighted *R*-factor wR and goodness of fit *S* are based on *F*^2^, conventional *R*-factors *R* are based on *F*, with *F* set to zero for negative *F*^2^. The threshold expression of *F*^2^ > σ(*F*^2^) is used only for calculating *R*-factors(gt) etc. and is not relevant to the choice of reflections for refinement. *R*-factors based on *F*^2^ are statistically about twice as large as those based on *F*, and *R*- factors based on ALL data will be even larger.

### Fractional atomic coordinates and isotropic or equivalent isotropic displacement parameters (Å^2^)

**Table:**

	*x*	*y*	*z*	*U*_iso_*/*U*_eq_	Occ. (<1)
C13	0.05834 (9)	0.8208 (3)	0.03041 (9)	0.0389 (4)	
H13	0.0302	0.7235	0.0424	0.047*	
O2	0.01845 (8)	1.1416 (2)	0.15941 (8)	0.0607 (5)	
C8	0.06408 (9)	0.9758 (2)	0.06998 (9)	0.0355 (4)	
O1	0.00415 (8)	0.8407 (2)	0.15781 (7)	0.0572 (4)	
C9	0.10590 (10)	1.1195 (3)	0.05077 (9)	0.0424 (5)	
H9	0.1096	1.2252	0.0763	0.051*	
C12	0.09496 (10)	0.8128 (3)	−0.02711 (9)	0.0418 (5)	
C14	0.02558 (9)	0.9872 (3)	0.13409 (9)	0.0397 (4)	
O4	0.12095 (11)	0.6430 (3)	−0.11917 (9)	0.0845 (6)	
N2	0.08814 (12)	0.6506 (3)	−0.07003 (10)	0.0655 (6)	
C11	0.13792 (10)	0.9514 (3)	−0.04684 (9)	0.0433 (5)	
H11	0.1629	0.9426	−0.0852	0.052*	
N3	0.18743 (11)	1.2557 (3)	−0.02654 (9)	0.0660 (6)	
C10	0.14210 (10)	1.1040 (3)	−0.00676 (9)	0.0441 (5)	
O5	0.21377 (11)	1.2487 (3)	−0.07970 (9)	0.0895 (7)	
N1	0.03402 (8)	0.4988 (2)	0.21019 (8)	0.0423 (4)	
H1A	0.0216	0.3954	0.1899	0.051*	
H1B	0.0188	0.5959	0.1880	0.051*	
H1C	0.0168	0.5001	0.2503	0.051*	
C5	0.14494 (11)	0.5352 (3)	0.15756 (10)	0.0464 (5)	
H5	0.1232	0.5520	0.1177	0.056*	
C4	0.10811 (10)	0.5068 (2)	0.21395 (9)	0.0378 (4)	
C1	0.24824 (11)	0.5139 (3)	0.22000 (10)	0.0443 (5)	
C3	0.13975 (11)	0.4821 (3)	0.27266 (10)	0.0504 (5)	
H3	0.1146	0.4624	0.3104	0.061*	
C6	0.21460 (11)	0.5381 (3)	0.16134 (10)	0.0501 (5)	
H6	0.2396	0.5568	0.1234	0.060*	
C2	0.20983 (11)	0.4868 (3)	0.27537 (10)	0.0527 (6)	
H2	0.2313	0.4713	0.3154	0.063*	
C7	0.32394 (11)	0.5136 (3)	0.22312 (13)	0.0601 (6)	
H7A	0.3382	0.5282	0.2676	0.072*	
H7B	0.3413	0.6137	0.1974	0.072*	
H7C	0.3408	0.3986	0.2064	0.072*	
O6	0.1864 (11)	1.4010 (15)	0.0066 (7)	0.079 (3)	0.59 (5)
O3	0.0382 (13)	0.549 (3)	−0.0614 (5)	0.086 (4)	0.59 (5)
O6'	0.2092 (15)	1.351 (5)	0.0179 (11)	0.090 (7)	0.41 (5)
O3'	0.0653 (14)	0.5080 (19)	−0.0405 (18)	0.086 (6)	0.41 (5)

### Atomic displacement parameters (Å^2^)

**Table:**

	*U*^11^	*U*^22^	*U*^33^	*U*^12^	*U*^13^	*U*^23^
C13	0.0403 (10)	0.0373 (10)	0.0390 (10)	0.0003 (8)	0.0010 (8)	0.0025 (8)
O2	0.0577 (10)	0.0609 (10)	0.0635 (10)	−0.0064 (7)	0.0159 (7)	−0.0254 (8)
C8	0.0338 (9)	0.0401 (10)	0.0327 (9)	0.0041 (7)	−0.0020 (7)	0.0007 (8)
O1	0.0631 (9)	0.0534 (9)	0.0552 (9)	0.0088 (7)	0.0223 (7)	0.0144 (7)
C9	0.0473 (11)	0.0409 (10)	0.0389 (10)	−0.0035 (8)	0.0004 (8)	−0.0036 (8)
C12	0.0492 (11)	0.0382 (10)	0.0380 (10)	0.0028 (8)	0.0002 (8)	−0.0046 (8)
C14	0.0317 (9)	0.0499 (12)	0.0374 (10)	0.0033 (8)	0.0000 (7)	−0.0025 (9)
O4	0.1219 (17)	0.0742 (12)	0.0575 (11)	−0.0063 (11)	0.0326 (11)	−0.0238 (9)
N2	0.0895 (16)	0.0515 (12)	0.0554 (12)	−0.0094 (11)	0.0180 (11)	−0.0127 (10)
C11	0.0471 (11)	0.0500 (11)	0.0327 (9)	0.0016 (9)	0.0043 (8)	0.0012 (8)
N3	0.0785 (14)	0.0701 (14)	0.0492 (11)	−0.0299 (11)	0.0148 (10)	−0.0049 (10)
C10	0.0443 (11)	0.0499 (12)	0.0382 (10)	−0.0098 (9)	0.0005 (8)	0.0027 (8)
O5	0.1144 (16)	0.0894 (14)	0.0647 (11)	−0.0438 (12)	0.0420 (11)	−0.0088 (10)
N1	0.0482 (10)	0.0403 (9)	0.0384 (9)	−0.0019 (7)	0.0036 (7)	−0.0038 (7)
C5	0.0577 (13)	0.0461 (11)	0.0356 (10)	0.0011 (9)	0.0045 (9)	0.0038 (8)
C4	0.0462 (11)	0.0303 (9)	0.0370 (10)	−0.0013 (7)	0.0048 (8)	−0.0041 (7)
C1	0.0465 (11)	0.0357 (10)	0.0506 (11)	−0.0015 (8)	0.0078 (9)	−0.0041 (8)
C3	0.0491 (12)	0.0672 (14)	0.0350 (10)	−0.0064 (10)	0.0089 (9)	−0.0023 (9)
C6	0.0572 (13)	0.0497 (12)	0.0434 (11)	−0.0016 (9)	0.0184 (10)	0.0024 (9)
C2	0.0507 (12)	0.0687 (15)	0.0387 (11)	−0.0048 (10)	0.0010 (9)	0.0003 (10)
C7	0.0497 (13)	0.0572 (14)	0.0735 (16)	−0.0016 (10)	0.0103 (11)	−0.0026 (12)
O6	0.116 (7)	0.063 (4)	0.058 (4)	−0.037 (4)	0.013 (4)	−0.006 (2)
O3	0.136 (9)	0.064 (5)	0.058 (3)	−0.053 (5)	0.016 (4)	−0.014 (3)
O6'	0.105 (10)	0.107 (13)	0.058 (6)	−0.068 (9)	0.016 (6)	−0.023 (7)
O3'	0.113 (9)	0.055 (4)	0.090 (12)	−0.017 (5)	0.034 (7)	−0.022 (5)

### Geometric parameters (Å, °)

**Table:**

C13—C12	1.384 (3)	N3—C10	1.475 (3)
C13—C8	1.389 (3)	N1—C4	1.469 (3)
C13—H13	0.9300	N1—H1A	0.8900
O2—C14	1.240 (2)	N1—H1B	0.8900
C8—C9	1.386 (3)	N1—H1C	0.8900
C8—C14	1.520 (3)	C5—C4	1.381 (3)
O1—C14	1.241 (2)	C5—C6	1.381 (3)
C9—C10	1.383 (3)	C5—H5	0.9300
C9—H9	0.9300	C4—C3	1.367 (3)
C12—C11	1.376 (3)	C1—C2	1.379 (3)
C12—N2	1.473 (3)	C1—C6	1.384 (3)
O4—N2	1.199 (2)	C1—C7	1.499 (3)
N2—O3	1.242 (12)	C3—C2	1.388 (3)
N2—O3'	1.279 (19)	C3—H3	0.9300
C11—C10	1.378 (3)	C6—H6	0.9300
C11—H11	0.9300	C2—H2	0.9300
N3—O5	1.208 (2)	C7—H7A	0.9600
N3—O6'	1.218 (16)	C7—H7B	0.9600
N3—O6	1.252 (12)	C7—H7C	0.9600
			
C12—C13—C8	119.15 (17)	C9—C10—N3	119.25 (18)
C12—C13—H13	120.4	C4—N1—H1A	109.5
C8—C13—H13	120.4	C4—N1—H1B	109.5
C9—C8—C13	119.31 (17)	H1A—N1—H1B	109.5
C9—C8—C14	120.25 (16)	C4—N1—H1C	109.5
C13—C8—C14	120.43 (16)	H1A—N1—H1C	109.5
C10—C9—C8	119.36 (18)	H1B—N1—H1C	109.5
C10—C9—H9	120.3	C4—C5—C6	118.84 (19)
C8—C9—H9	120.3	C4—C5—H5	120.6
C11—C12—C13	122.91 (18)	C6—C5—H5	120.6
C11—C12—N2	117.56 (18)	C3—C4—C5	120.86 (19)
C13—C12—N2	119.53 (18)	C3—C4—N1	119.86 (16)
O2—C14—O1	124.57 (18)	C5—C4—N1	119.26 (17)
O2—C14—C8	117.84 (17)	C2—C1—C6	117.8 (2)
O1—C14—C8	117.58 (17)	C2—C1—C7	121.0 (2)
O4—N2—O3	121.6 (4)	C6—C1—C7	121.17 (19)
O4—N2—O3'	123.5 (6)	C4—C3—C2	119.30 (18)
O3—N2—O3'	34.5 (6)	C4—C3—H3	120.3
O4—N2—C12	119.18 (19)	C2—C3—H3	120.3
O3—N2—C12	117.2 (6)	C5—C6—C1	121.78 (18)
O3'—N2—C12	113.2 (12)	C5—C6—H6	119.1
C12—C11—C10	116.52 (18)	C1—C6—H6	119.1
C12—C11—H11	121.7	C1—C2—C3	121.4 (2)
C10—C11—H11	121.7	C1—C2—H2	119.3
O5—N3—O6'	122.9 (8)	C3—C2—H2	119.3
O5—N3—O6	122.1 (6)	C1—C7—H7A	109.5
O6'—N3—O6	29.2 (15)	C1—C7—H7B	109.5
O5—N3—C10	118.59 (19)	H7A—C7—H7B	109.5
O6'—N3—C10	115.5 (11)	C1—C7—H7C	109.5
O6—N3—C10	117.9 (7)	H7A—C7—H7C	109.5
C11—C10—C9	122.73 (18)	H7B—C7—H7C	109.5
C11—C10—N3	118.01 (18)		

### Hydrogen-bond geometry (Å, °)

**Table:**

*D*—H···*A*	*D*—H	H···*A*	*D*···*A*	*D*—H···*A*
N1—H1A···O2^i^	0.89	1.94	2.803 (2)	163
N1—H1B···O1	0.89	1.90	2.761 (2)	163
N1—H1C···O2^ii^	0.89	2.22	3.045 (2)	153
N1—H1C···O1^ii^	0.89	2.24	3.030 (2)	147
C3—H3···O1^ii^	0.93	2.59	3.344 (3)	138
C13—H13···O3^iii^	0.93	2.43	3.351 (7)	173

Symmetry codes: (i) *x*, *y*−1, *z*; (ii) −*x*, *y*−1/2, −*z*+1/2; (iii) −*x*, −*y*+1, −*z*.
